# Supplementary material for: Cost of fruiting, size regulation, and senescence in a long-lived geophyte
Source: PLoS One. 2026 May 8;21(5):e0347038. doi: 10.1371/journal.pone.0347038 (PMC13155616; doi:10.1371/journal.pone.0347038)
Supplement: Attachment — Submitted filename: Response to Reviewers 2Feb.docx [file pone.0347038.s002.docx]

Response to Reviewers

PONE-D-25-47577

Cost of fruiting, size regulation, and senescence in a long-lived geophyte

Pasted-in comments by the editor and three reviewers are in black. Comments by James Thomson are interpolated in red. The revised MS with Track Changes shown and the clean version with Track Changes Accepted are uploaded as two separate files. Please note that I made a few late changes that are not shown in the version with Track Changes shown. These concern formatting rather than substance.

Most of the editorial comments appear to be restatements of comments attributed to the reviewers. I treat those only briefly in the first section. I answer them more fully below, in the sections containing the reviewers’ comments. My responses under Reviewer 1 include a critical explanation of why the unusual nature of the 33-year data set requires a partially unusual organizational approach.

I have been trying to wrap up this revision while confined to hospital awaiting a triple bypass operation tomorrow (3 Feb). Conditions in my shared room make it hard to concentrate, and the Wi-Fi is weak. As I am uploading new pieces under Attach Files, EM tells me to delete old ones, but I don’t see how to delete them or even how to select them. Therefore, some chunks have been superseded by newer ones. If two versions are present , the one with the newer data should be selected. Some shortcomings of format might remain, but I hope that the submitted documents are good enough for you to initiate the review while I am indisposed. I will happily work to make further adjustments after I have some time to recover. This is the best that I can do for now. Thanks for your consideration.

1.Please ensure that your manuscript meets PLOS ONE's style requirements, including those for file naming. The PLOS ONE style templates can be found at

<https://journals.plos.org/plosone/s/file?id=wjVg/PLOSOne_formatting_sample_main_body.pdf> and

[https://journals.plos.org/plosone/s/file?id=ba62/PLOSOne_formatting_sample_title_authors_affiliations.pdf](https://can01.safelinks.protection.outlook.com/?url=https%3A%2F%2Ftrack.editorialmanager.com%2FCL0%2Fhttps%3A%252F%252Fjournals.plos.org%252Fplosone%252Fs%252Ffile%253Fid%3Dba62%252FPLOSOne_formatting_sample_title_authors_affiliations.pdf%2F1%2F010f0199c2365f65-8a20264a-735b-4da6-a186-45f08b68e7ea-000000%2F4WpGgHjtleDaNR7PltKMGeRsXrXo-oWloO-hUadbzIY%3D232&data=05%7C02%7Cjames.thomson%40utoronto.ca%7Cb19a11841e4a44e3d7c508de062863f8%7C78aac2262f034b4d9037b46d56c55210%7C0%7C0%7C638954968006821179%7CUnknown%7CTWFpbGZsb3d8eyJFbXB0eU1hcGkiOnRydWUsIlYiOiIwLjAuMDAwMCIsIlAiOiJXaW4zMiIsIkFOIjoiTWFpbCIsIldUIjoyfQ%3D%3D%7C0%7C%7C%7C&sdata=lC00f4cyfVgdC7i5wlrom1JEkYVsOmnqBDIikW1rVpk%3D&reserved=0)

I believe that I have made the necessary changes.

2. We noted in your submission details that a portion of your manuscript may have been presented or published elsewhere ”Data have been placed in a public archive. Thomson, J.D. 2025. Life histories of the perennial geophyte Erythronium grandiflorum (Liliaceae) in Colorado subalpine transplant garden from annual measurements, 1991 onward ver 3. Environmental Data Initiative. [https://doi.org/10.6073/pasta/e3e67039141f54c24c87326e5d15f49a”](https://doi.org/10.6073/pasta/e3e67039141f54c24c87326e5d15f49a%E2%80%9D) Please clarify whether this [conference proceeding or publication] was peer-reviewed and formally published. If this work was previously peer-reviewed and published, in the cover letter please provide the reason that this work does not constitute dual publication and should be included in the current manuscript. The cited location is for a data repository, not a publication. See below under point 3.

3. Thank you for uploading your study's underlying data set. Unfortunately, the repository you have noted in your Data Availability statement does not qualify as an acceptable data repository according to PLOS's standards.

Points 2 and 3 both concern the reason why this revision has taken so long. As I protested in correspondence with several editorial assistants, I was convinced that omitting the Environmental Data Initiative from your list of suitable data repositories was incorrect and should be corrected. I was not about to abandon EDI, where my data sets have been posted for several years. Therefore, I set this paper aside until you made the correction. I never got a response from PLOSONE, but EDI tells me that you have in fact now added EDI to the list of acceptable repositories. Thus, this issue has become moot.

At this time, please upload the minimal data set necessary to replicate your study's findings to a stable, public repository (such as figshare or Dryad) and provide us with the relevant URLs, DOIs, or accession numbers that may be used to access these data. For a list of recommended repositories and additional information on PLOS standards for data deposition, please see [https://journals.plos.org/plosone/s/recommended-repositories](https://can01.safelinks.protection.outlook.com/?url=https%3A%2F%2Ftrack.editorialmanager.com%2FCL0%2Fhttps%3A%252F%252Fjournals.plos.org%252Fplosone%252Fs%252Frecommended-repositories%2F1%2F010f0199c2365f65-8a20264a-735b-4da6-a186-45f08b68e7ea-000000%2FO2S6CJuI4ZIvzVBvP98ElfY-7aAfgjUCsE0vQdWLflk%3D232&data=05%7C02%7Cjames.thomson%40utoronto.ca%7Cb19a11841e4a44e3d7c508de062863f8%7C78aac2262f034b4d9037b46d56c55210%7C0%7C0%7C638954968006855815%7CUnknown%7CTWFpbGZsb3d8eyJFbXB0eU1hcGkiOnRydWUsIlYiOiIwLjAuMDAwMCIsIlAiOiJXaW4zMiIsIkFOIjoiTWFpbCIsIldUIjoyfQ%3D%3D%7C0%7C%7C%7C&sdata=vcb4Wr7dYHzlyIvywp5cYP3T2G3T6Ej%2FCCbYdAynpy4%3D&reserved=0).

Done.

4. If the reviewer comments include a recommendation to cite specific previously published works, please review and evaluate these publications to determine whether they are relevant and should be cited. There is no requirement to cite these works unless the editor has indicated otherwise.

Done, further explanation below.

Additional Editor Comments:

Following an exhaustive review of the manuscript on the life cycle of Erythronium grandiflorum by three reviewers, crucial aspects requiring attention have been identified to significantly improve the scientific quality and impact of the work. The current manuscript, while containing valuable data based on years of research, needs substantial restructuring to meet scientific publication standards.
The primary concern focuses on the document's structure, which currently resembles an essay more than a formal scientific article. It is essential to incorporate clearly defined sections for Methodology, Statistical Analysis, and Discussion. Results and discussion should be separated to improve clarity and logical flow. The writing style should remain consistent, using past tense for reporting results and maintaining a formal tone throughout the document. Done or addressed below.
From a technical perspective, the manuscript would benefit significantly from greater integration with recent studies on physiological and biochemical mechanisms. It is particularly important to relate the observed cost of fruiting (8% reduction in corm weight per fruit) to changes in photosynthetic pigments, antioxidant enzyme activities, and hormonal balance, especially concerning gibberellic acid and cytokinin. Including the suggested reference from Khan and Nabi (2024) would strengthen this connection between morphological results and underlying biochemical mechanisms.

The request to turn a demographic study into a physiological/biochemical one is both odd and impossible. I have a huge demographic data set but no physiological data whatsoever. More below, under Rev. 2.
A crucial aspect requiring clarification is the distinction between identified senescence pathways: gradual decline versus sudden collapse due to fungal attack. It is recommended to include biochemical or anatomical markers to support claims about "classical senescence." Additionally, the interpretation of "fungal attacks" should be approached with caution, considering that artificial study conditions might have altered soil microorganism balance. I have no biochemical markers and no anatomical data other than the corm weights that form the basis for my analysis of growth trajectories. I have tried to make it even more obvious that the soil in the garden has necessarily received additions of potting soil and cannot be viewed as “natural.” It’s impossible to exhume corms without disturbing soil! My design at least pools soil from numerous pots to homogenize conditions.
Regarding visual presentation, figures need improvement, especially Figure 5, which requires confidence intervals and clearer labels, and Figure 6, where plants corresponding to gradual versus sudden death should be specified. Please see general comments on statistics, below. I have amplified the figure labels, but confidence intervals are inappropriate for Figure 5. Terminology should be consistent throughout the document, avoiding colloquial phrases and maintaining precise scientific language. I have made adjustments.
The manuscript should also address climate change implications, considering how changing frost patterns and pollination windows could affect the cost-benefit balance of reproduction. This is a very large question that I am pursuing with other collaborators. Progress is limited by their speed. The data here are valuable and noteworthy in their own right. The importance of long-term monitoring in validating evolutionary life history theories should be highlighted, citing fundamental works such as Williams (1957) and Obeso (2002). Done.
Finally, a critical revision of redundant or unnecessary sections is recommended, retaining only those examples and discussions that directly contribute to the main scientific argument. Methodology should be described concisely, referencing previous work where appropriate, and new data should be clearly differentiated from previously published results. Done (and see below).
With these modifications, the manuscript has the potential to become a significant contribution to the field, making the most of the valuable dataset compiled during years of dedicated research. I hope so.

Reviewer #1: The manuscript submitted for review focuses on the fascinating subject of the life cycle of Erythronium grandiflorum. The author has devoted many years to this topic and has already published several papers on it. Research into the biology of species is very difficult and often requires special, 'original' methodology. Unfortunately, interpreting the data is not easy. While the manuscript undoubtedly contains valuable data, it requires thorough rewriting and adaptation to the editorial requirements of PLOS ONE. Features that the reviewer considers unconventional arise partly from the two-fold nature of the paper. Primarily, it is a conventional report that asks and answers a specific pair of life-history questions: Do these plants regulate body size, and does the cost of fruiting play a role in this? The data adequately answer these questions. Secondarily, I want to publicize the data set and the study in the hopes of recruiting new investigators to continue studying other questions that are not yet solved. Here, I highlight the question of whether these plants undergo senescence, and if so, in what manner? Not enough plants have died yet to settle these questions, so my discussion of these points is necessarily more speculative. I raise hypothetical possibilities to elicit reader interest and to argue for keeping the study going. This gives this portion an unconventional quality that the reviewer found off-putting. I have tried to make the paper more conventional, but I consider it essential to address the nature of senescence. For most plant demographers, that would be the most interesting aspect of the data set.
The present form of the text is more in the style of an essay than a professional scientific paper. I address this style question above; nevertheless, I have provided explicitly labeled Methods, Results and Discussion sections as requested. A section entitled 'Materials and Methods' should be added that concisely describes the research methodology without going into too much detail and refers to previously published works in which I believe the methodology was the same. The reviewer has invented a fundamental misunderstanding. Previous publications concerned ecological questions in the field population, *not* demographic studies in the transplant garden. If previous papers overlapped with this one, I would have said so. Yes, the study organism and the study site are the same, but the questions and the data are completely different. I have aimed to describe the methodology in enough detail to clarify the nature and the limitations of the data. I provide further detail in the EDI archive. Many of the paragraphs in the paper are digressions and should be included in the Discussion section. I have tried to reduce digressions. I have also labeled as “Discussion” some parts that the reviewer probably saw as digressive. I am also interested in how the data presented in this manuscript differs from the results of previously published studies conducted over a shorter period of time. Previous papers concern utterly different questions with utterly different data sets.
I'm wondering whether the longer research period allowed for new data to be established, or if it simply confirmed what was already known. This continues the misunderstanding explained above. I would also like to suggest to the author that he interprets the results of his research into ‘fungal attacks’ with a little more caution, given that he conducted his research under artificial conditions, which could have disturbed the balance of soil microorganisms. In their natural state, soil microorganisms exist within a balanced ecosystem that comprises both fungi and bacteria. Some of these microorganisms, such as bacteria, are known to stimulate plant growth and development; however, under disturbed conditions, they can cause plants to become weak and die. Some bacteria can increase a plant's susceptibility to fungal infections. Since the author exhumed the corms, he may have affected their ‘quality of life’ in this way. I would therefore exercise caution when interpreting the obtained results. All of this is true, but that does not render the observations meaningless. Observations of the condition of underground structures could not be made without disturbing the soil, and those disturbances were conducted following strict, consistent protocols over the years of the study. I thought that this point was obvious, so I did not stress it. I have now conceded this point more explicitly. (I do wonder what evidence the reviewer has for the assertion that the soil ecosystem is “balanced.” I doubt it, although without an operational definition of “balanced,” the statement is meaningless.)
Minor:
Lines 51–52: This sentence is unnecessary. See immediately below.
Lines 56–57: Is this sentence necessary? This information is *essential* to the paper’s goal of recruiting new investigators. It needs to appear early in the paper; otherwise, potential readers may not get to it. Furthermore, PLOS ONE requires data archiving. As stated here, the archive provides additional information that can be consulted to clarify any matters that a reader is not sure about.
Lines 94–102: This section should be shortened and the digressions omitted. Section removed.
Lines 117–128: Is this section necessary? I guess not. I wrote it to explain why I did not start the study plants from seed. Given that the study’s goal evolved from genetics to demography, this was a poor decision that I wanted to justify. Although it hurts my pride, I have removed it.
Lines 144–149: As above, are these examples necessary? Yes. I concede that these lines may appear to be an overly detailed digression to a superficial reader who never consults the EDI archive, but it is absolutely essential for anyone who wants to understand how the plant IDs incorporate genetic identity and splitting history. Without this key information, those IDs would appear as mystifying gobbledegook. (This reinforces my inference that none of the reviewers tried to consult the data archive.)

Reviewer #2: ***The discussion could benefit from broader integration with recent studies that examine physiological and biochemical mechanisms underlying fruiting costs and senescence.
Please relate the observed cost of fruiting (8% reduction in corm weight per fruit) to changes in photosynthetic pigments, antioxidant enzyme activities, and hormonal balance (especially gibberellic acid and cytokinin), which are known to influence resource allocation and longevity.
With all due respect, this review’s recommendations are completely irrelevant to my study. They are also impossible for me to comply with. I have made no measurements of the physiological and biochemical factors that interest this reviewer. Furthermore, removing tissue to make those measurements would invalidate the demographic imperative of following intact plants through time.
*** Strongly recommended citation:
Khan, M. N., & Nabi, G. (2024). The potential role of gibberellic acid in regulating photosynthetic pigments, fruit quality and antioxidant enzymes in sweet lime Citrus limetta Risso. Pakistan Journal of Botany, 57(2).
This paper would help the authors connect morphological outcomes (size regulation, senescence) to underlying biochemical mechanisms (GA-mediated regulation, oxidative stress mitigation), offering a more holistic perspective on plant life-history trade-offs.
I have read the abstract of this study and find zero applicability to my paper. The authors applied experimental treatments of GA to lime plants and noted various responses. It may be a fine study, but it comes from a different universe. It has nothing to do with demography or life-history.
***The paper suggests two senescence pathways (gradual decline vs. sudden collapse due to fungal attack). Please differentiate physiological senescence from pathological death more explicitly. I have revised this section to highlight the factors that distinguish these two pathways to death. However, going too far in this direction would run afoul of Rev. 1’s reservations.

If possible, consider including or referencing biochemical or anatomical markers (e.g., tissue browning, ROS accumulation, chlorophyll degradation) to strengthen claims of "classical senescence." Not possible; aside from the occasional lesions that I discuss, I have no such data. There is no visible chlorophyll in the underground corms.

***Ensure Figure 5 (probability of trauma vs. corm weight) is clearly labeled and includes confidence intervals or error bars. See my comments elsewhere about statistics.

In Figure 6, specify which plants correspond to gradual vs. sudden death to guide interpretation. I have tried to clarify this; I have also more explicitly directed interested readers to the voluminous output in the archive. This includes growth trajectories for hundreds of individuals. It would be silly to think of including this material in a paper for PLOS ONE.

Please confirm that all figures referenced in the text (Figs. 1–7) are included and legible.

***Replace phrases like “plants might grow themselves to death” with more formal wording such as “plants may experience mortality due to overaccumulation of biomass and subsequent vulnerability to infection.” I disagree that the suggested rewording would be an improvement, but I have revised the offending phrase.

Ensure consistent use of terms like “cost of fruiting” vs. “reproductive cost.” I have tried to clarify that I use the former as a specific term with an operational definition derived from corm weights; the latter is a more general umbrella concept in evolutionary biology.

***Line 17–19: consider rephrasing to “producing one fruit results in approximately 8% reduction in potential corm weight gain.” OK.

Line 199–201: clarify speculative statements with references or indicate explicitly as a hypothesis. OK.

***Briefly discuss potential climate change implications, as shifting frost patterns and pollination windows may alter the cost-benefit balance of reproduction. This is a fine suggestion for an additional paper, but is beyond the scope of what I can do here. I have agreed to collaborate on such a paper with David Inouye, who has compiled extensive climate data for the study locality. This will not be a quick process.

Mention the importance of long-term monitoring in validating evolutionary life-history theories (Williams 1957, Obeso 2002). Done.

Reviewer #3: The manuscript titled "Cost of fruiting, size regulation, and senescence in a long-lived geophyte" addresses a very interesting topic—the study of senescence in plants. The main issue with the current version is that its structure does not conform to a standard scientific format. It lacks dedicated sections for Methods, Statistical Analysis, and Discussion. Furthermore, the statistical analyses need to be described more clearly. Currently, the results and discussion are intertwined, which reduces the clarity of the presentation. This frustratingly brief review is solely concerned with style rather than substance. There is in fact no evidence that this reviewer grappled with any scientific content in the submission at all. The reviewer seems convinced that there is one single universal organizational template to which all publications must conform. As explained in my response to Rev. 1, the paper’s organization is unconventional by design: the study itself is unconventional, and virtually unique. Furthermore, my goal of recruiting other researchers to inherit the study is unconventional. The reviewer is welcome to his or her opinion, of course, but I don’t think that an absolutely conventional paper could accomplish my purpose. I see this as an issue for the discretion of an editor. Perhaps there is no room in PLOS ONE for this report, but I hope that my revision has made my approach more conventional and acceptable.

Additionally, the writing style should be made consistent. The reporting of results should be in the past tense, whereas the current narrative often resembles informal notes rather than a formal scientific paper. I have gone through the text with this point in mind, and have generally tried to put my account into a stiff collar rather than work clothes. That said, the combination of tenses often reflects that fact that the study is still going on. Some statements concern specific events that have happened in the past; others reflect ongoing processes that continue the shape demographic trajectories. These distinctions are important in my mind, and I tried to be scrupulous in distinguishing them. I fear that the reviewer’s concern reflects a superficial reading of the MS. Nevertheless, I have reviewed verb tenses throughout and made changes where clarity was in doubt.

Regarding this reviewer’s request to clarify the statistics: Given that the paper is essentially a summary of a huge and rather unwieldy data set, stats are obviously critical. For this reason, I entrusted the analysis to a colleague with impeccable credentials. David Andrews has chaired the Statistics Department at the University of Toronto, has been president of the Statistical Society of Canada, and ran the U of T’s statistical consulting service for many years. He has also visited my study site, and we have had long discussions about the biology and the data. He designed all of the analyses in the paper, wrote the R code, and prepared the figures. The EDI archive contains further explanations and additional output, as well as the R code for anyone who cares to probe more deeply. It’s obvious that Rev. 3 did not consult the EDI archive. (It doesn’t appear that the other reviewers did either.) As a self-effacing emeritus professor with nothing to gain by another publication, David declined my offer of co-authorship on the previous draft. However, given that one of the three reviewers seemed sceptical, I have prevailed upon David to join me as an author on the revision. I hope that readers who lack the motivation or ability to plow through the R code will be sufficiently reassured by his imprimatur.

Despite these issues, the paper has significant potential but requires substantial reorganization and stylistic refinement to meet the standards of a scientific publication.

[NOTE: If reviewer comments were submitted as an attachment file, they will be attached to this email and accessible via the submission site. Please log into your account, locate the manuscript record, and check for the action link "View Attachments". If this link does not appear, there are no attachment files.]

While revising your submission, please upload your figure files to the Preflight Analysis and Conversion Engine (PACE) digital diagnostic tool, [https://pacev2.apexcovantage.com/](https://can01.safelinks.protection.outlook.com/?url=https%3A%2F%2Ftrack.editorialmanager.com%2FCL0%2Fhttps%3A%252F%252Fpacev2.apexcovantage.com%252F%2F1%2F010f0199c2365f65-8a20264a-735b-4da6-a186-45f08b68e7ea-000000%2FYee-6nDBL3gKRDXbEU7Q1MhJyHF_6gNDkvSXLBE5BVM%3D232&data=05%7C02%7Cjames.thomson%40utoronto.ca%7Cb19a11841e4a44e3d7c508de062863f8%7C78aac2262f034b4d9037b46d56c55210%7C0%7C0%7C638954968006936264%7CUnknown%7CTWFpbGZsb3d8eyJFbXB0eU1hcGkiOnRydWUsIlYiOiIwLjAuMDAwMCIsIlAiOiJXaW4zMiIsIkFOIjoiTWFpbCIsIldUIjoyfQ%3D%3D%7C0%7C%7C%7C&sdata=LmkKiT7gzdRIkyNGWr5Tpa2UujX4w%2BXgDBMsAs8R3fY%3D&reserved=0). PACE helps ensure that figures meet PLOS requirements. To use PACE, you must first register as a user. Registration is free. Then, login and navigate to the UPLOAD tab, where you will find detailed instructions on how to use the tool. If you encounter any issues or have any questions when using PACE, please email PLOS at [figures@plos.org](mailto:figures@plos.org). Please note that Supporting Information files do not need this step.

I went to the PACE site, watched the ad video, and tried to establish a free account as directed. I was unable to do so, probably for inexplicable reasons that have always hampered my use of Google functions. I believe that the current figures are good enough for you to decide whether to accept the paper or not. If they need further refinement, I’d prefer to address that if you accept the paper. I beg to have this paper enter your evaluation process before my heart surgery tomorrow I can’t view fussing with a refractory PACE account as a suitable use of my limited time. I hope that you can cut me some slack on this point.

Sincerely,

James Thomson
